# Modulating T Cell Responses via Autophagy: The Intrinsic Influence Controlling the Function of Both Antigen-Presenting Cells and T Cells

**DOI:** 10.3389/fimmu.2018.02914

**Published:** 2018-12-14

**Authors:** Seth D. Merkley, Cameron J. Chock, Xuexian O. Yang, James Harris, Eliseo F. Castillo

**Affiliations:** ^1^Clinical and Translational Science Center, University of New Mexico Health Sciences Albuquerque, NM, United States; ^2^Department of Molecular Genetics and Microbiology, University of New Mexico Health Sciences Albuquerque, NM, United States; ^3^Autophagy Inflammation and Metabolism Center of Biomedical Research Excellence, University of New Mexico Health Sciences Albuquerque, NM, United States; ^4^Rheumatology Group, Centre for Inflammatory Diseases, Department of Medicine, School of Clinical Sciences at Monash Health, Faculty of Medicine, Nursing and Health Sciences, Monash University Clayton, VIC, Australia; ^5^Division of Gastroenterology and Hepatology, Department of Internal Medicine, University of New Mexico School of Medicine Albuquerque, NM, United States

**Keywords:** autophagy, T cells, macrophages, inflammation, immunometabolism, immunotherapy

## Abstract

Autophagy is a homeostatic and inducible process affecting multiple aspects of the immune system. This intrinsic cellular process is involved in MHC-antigen (Ag) presentation, inflammatory signaling, cytokine regulation, and cellular metabolism. In the context of T cell responses, autophagy has an influential hand in dictating responses to self and non-self by controlling extrinsic factors (e.g., MHC-Ag, cytokine production) in antigen-presenting cells (APC) and intrinsic factors (e.g., cell signaling, survival, cytokine production, and metabolism) in T cells. These attributes make autophagy an attractive therapeutic target to modulate T cell responses. In this review, we examine the impact autophagy has on T cell responses by modulating multiple aspects of APC function; the importance of autophagy in the activation, differentiation and homeostasis of T cells; and discuss how the modulation of autophagy could influence T cell responses.

## Introduction

Autophagy is an evolutionarily conserved cellular response that can selectively or non-selectively direct cargo to the lysosome where cargo is degraded for subsequent recycling (1). Autophagy is upregulated under conditions of physiological stress, particularly amino acid starvation, during which it acts as a cell survival mechanism, recycling cytoplasmic macromolecules. The term “autophagy” encompasses three complementary processes; microautophagy, chaperone-mediated autophagy (CMA) and macroautophagy. Each form of autophagy involves the delivery of cytoplasmic substrates to lysosomes for degradation. Microautophagy involves the direct engulfment of cytoplasmic cargo by lysosomes (or the vacuole in plants and fungi) and can either be non-selective, or selective, as in the cases of micropexophagy (degradation of peroxisomes), micromitophagy (degradation of mitochondria), and piecemeal microautophagy of the nucleus (PMN) (2). CMA involves the selective targeting of soluble cytosolic proteins to lysosomes for degradation. Proteins targeted by CMA have a common pentapeptide motif (KFERQ) which is recognized by the chaperone protein Hsc70 (heat shock cognate protein of 70 KDa) (3). Macroautophagy involves the formation of double-membrane vesicles, termed autophagosomes, around a portion of cytoplasm, including organelles such as damaged/leaky mitochondria (e.g., mitophagy), misfolded or aggregated proteins (e.g., aggrephagy) or microbes (e.g., xenophagy) (4). Autophagosomes are then able to fuse with functional lysosomes (“maturation” or “flux”) for degradation of the luminal contents.

Macroautophagy (herein, called autophagy) is a fundamental, homeostatic process integrated into both the innate and adaptive arms of the immune system (4, 5). The immunological functions of autophagy appear to interact and modify “classical” innate immune pathways including (i) direct microbial elimination; (ii) cooperation with pattern recognition receptors (PRR); (iii) inflammasome regulation; (iv) secretion of biomolecules; and (v) major histocompatibility complexes (MHC) and antigen processing (4–6). In addition, autophagy plays a major role in B and T cell activation, proliferation and survival as well as affects positive and negative thymic selection of naïve T cells (7, 8). This integration points to autophagy as an important cellular process that safeguards the host with a functional innate and adaptive immune system. It also highlights how perturbations in autophagy, either because of genetics or environmental factors, might influence health and disease. Notwithstanding, autophagy is an attractive therapeutic target to modulate T cell responses directly in T cells or indirectly through APC like dendritic cells (DC) and macrophages. This network of communication between autophagy with the immunological pathways as well as cellular metabolism is discussed in the following few sections with an emphasis on how it affects T cell responses.

## Crosstalk Between Autophagy and Innate Pathways to Influence T Cell Responses

The above immunological functions of autophagy represent the interplay and interaction of this cellular process within a single cell (e.g., pathogen elimination in APC; proliferation through clearance of cell cycle proteins in T cells). This section seeks to discuss the physiological and immunological relevance of autophagy and how it interacts at a systemic level—whereby autophagy in innate immune cells (e.g., DC and macrophages) affects CD8^+^ and CD4^+^ T helper (TH) cell responses. This type of crosstalk may play a critical role in the pathogenesis of various diseases such as Crohn's disease (CD), rheumatoid arthritis (RA) and multiple sclerosis (MS) as well as chronic infections like tuberculosis (TB).

### Autophagy and Antigen Presentation

MHC-restricted antigen presentation is key to the specificity of immunity and marks an important intersection between innate and adaptive immune pathways. Processed antigenic peptides are loaded on Class I or II MHC molecules and presented by professional APC to T cells for priming and activation. MHC class I-restricted antigens, which stimulate primed CD8^+^ T cells, originate mostly from endogenous proteins, processed by proteasomes and transported to the ER for loading (9). MHC Class II-restricted antigens, on the other hand, are recognized by CD4^+^ T cells and were originally thought to be almost exclusively of extracellular origin, taken up by endocytosis, micropinocytosis, or phagocytosis and processed by specialized endosomal and lysosomal enzymes. However, some exogenous antigens can be cross-presented by MHC Class I (MHC I) molecules, while MHC Class II (MHC II) molecules can also present endogenous proteins (9).

**Figure 1:**
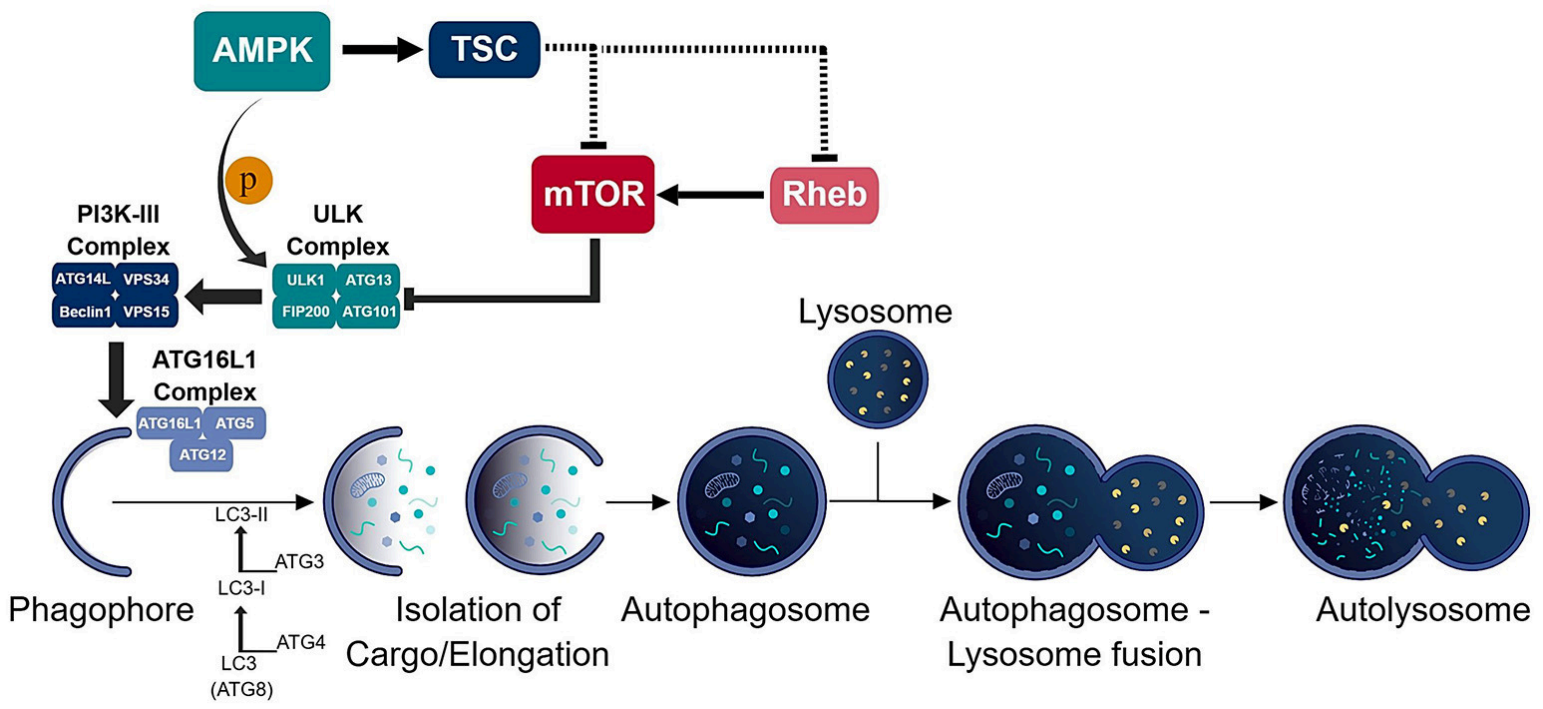
Schematic of the autophagic process. The consecutive activation of the ULK (ULK1/ATG13/FIP200/ATG101) and PI3K-III (ATG14L/VPS34/Beclin-1/VPS15) complexes results in the generation of the phagophore and recruitment of the ATG16L1 (ATG16L1-ATG5-ATG12) conjugation complex to the phagophore. ATG16L1 complex initiates the elongation of the phagophore to become an autophagosome. The formation of the autophagosome and isolation of cargo is assisted by the LC3 (ATG8) conjugation system. The isolated cargo is then degraded in the autolysosome which is formed via autophagosome-lysosome fusion.

Autophagy is an intricate pathway involving numerous proteins to execute several steps that lead to the degradation of biomolecules, organelles and pathogens (Figure 1). A number of studies indicate that autophagy represents an important factor in the presentation of endogenous antigens via MHC II (10). Particularly, autophagosomes have been shown to fuse directly with MHC II loading compartments (11, 12). Moreover, fusion of viral and tumor antigens to the ATG8 (autophagy-related-gene 8) family protein LC3-II, which localizes to autophagosomal membranes, increases presentation to CD4^+^ T cells (12–14). Interestingly, an unbiased study of the MHC II ligandome by mass spectrometry, revealed nutrient starvation (a mechanism to induce autophagy) leads to a 50% increase in the presentation of nuclear and cytosolic antigens (15). Many MHC II ligands express Atg8-binding domains (LC3-interacting regions; LIR) that may target them to autophagosomes (15). As it is, functional autophagy has been shown to facilitate presentation of several pathogen-derived antigens, including nuclear antigen 1 of the Epstein-Barr virus, the mycobacterial antigen Ag85B and modified vaccinia virus Ankara encoded antigens (16–18).

Autophagy also appears to play an important role in thymic selection of T cells, mediated by thymic epithelial cells (TEC). TEC express low levels of peripheral tissue-specific antigens under the transcriptional regulation of the autoimmune regulator (AIRE), which requires loading of endogenous antigens onto MHC II molecules for positive and negative selection (19). Nedjic and colleagues revealed TEC use autophagy to load endogenous molecules to MHC II molecules and interference of autophagy in TEC results in colitis and multi-organ inflammation (20). They further show positive selection of some, but not all, transgenic CD4^+^ thymocytes is inhibited in *Atg5*^−/−^ thymi (20). In addition, targeting of cognate antigens to autophagosomes by fusing them with either LC3B or mitochondria results in negative selection of the respective TCR transgenic CD4^+^ T cells (21).

A role for autophagy in MHC I presentation has been controversial. MHC I presentation is dependent on proteasomal processing of antigenic peptides and is dependent on the transport of peptides from endosomal compartments to the cytosol, the very opposite of autophagy, which transports cytosolic components to endocytic compartments. Nevertheless, studies have indicated roles for autophagy in this antigen presenting pathway. Loss of autophagy can redirect autophagy substrates to proteasomes for processing and MHC I-restricted presentation (22). Furthermore, autophagosomes have been shown to target proteasome components for degradation (23, 24). Conversely, inhibition of normal MHC I processing pathways, which can occur in some viral infections, may result in increased endosomal/autophagosomal involvement (10). Autophagy has also been implicated in cross-presentation of exogenous antigens. Of note, mouse CD8^+^ DC, which are the predominant cross-presenters *in vivo* (25, 26), have higher basal autophagy rates than CD8^−^ DC, which are not capable of cross-presentation (27). Similarly, both mouse and human DC more adept at cross-presentation of accumulated large ubiquitinated aggregates, also termed dendritic cell aggresome-like structures (DALIS), that can function as reservoirs for MHC I antigens (22), and these structures were also positive for the autophagy receptor p62/SQSTM1. Additionally, LC3 was recruited to zymosan-containing phagosomes in these cells, indicating that the autophagy machinery intersects with phagosomes containing exogenous antigens. This study went on to demonstrate that the contribution of autophagy was dependent on the form of antigen, being required for cross-presentation of soluble antigen (ovalbumin, OVA), but not OVA targeted to apoptotic bodies or the receptor DEC-205 (cell-associated antigen) (27). How autophagy regulates the cross-presentation of soluble antigens is not yet clear and requires further elucidation. Nonetheless, these studies support an underappreciated role for autophagy in MHC I presentation; however, the effect it has on CD8^+^ T cell responses remains unclear.

The intersection of autophagy with both MHC I and II pathways reiterate the importance of autophagy in innate cells in controlling T cell responses. Interestingly, MHC II molecules show the strongest linkage to inflammatory and autoimmune diseases like CD, MS, RA, systemic lupus erythematosus (SLE) and type 1 diabetes (T1D) (28, 29). Genome-wide association studies have also implicated autophagy genes *Atg5* and *Atg16l1* in the susceptibility of SLE and CD, respectively (30–34). It is unclear if this genetic linkage is tied to direct autophagy-MHC crosstalk, defects in autophagy, or hyperactive autophagy. However, it was recently shown in animal model of MS that the autophagy gene *Atg5* was required in DC to present endogenous self-peptides to autoreactive CD4^+^ T cells (35). ATG5 assisted in the fusion of phagosomes containing injured oligodendroglial cells with MHC II compartments. In the absence of ATG5, there was a decrease in autoreactive CD4^+^ T cells which delayed the onset of disease and reduced clinical severity compared to mice expressing ATG5 in DC (35). Given the apparent role of autophagy in central tolerance, a mechanism to limit autoreactive T cells (20, 21), it is plausible to link autophagy to autoreactive T cells. Thus, while full mechanistic understanding of autophagy/autophagy genes and MHC I and II pathways remains elusive, it is evident autophagy in APC can greatly influence T cell responses via both MHC I and II pathways.

### Autophagy and IL-1 Family Cytokines

Numerous studies have shown autophagy intersects with the production, processing and release of IL-1 family cytokines (36, 37). Loss of autophagy in macrophages and DC results in the increased release of IL-1β and IL-18 in response to Toll like receptor (TLR)3 and TLR4 ligation. This is dependent on Toll/IL-1 receptor domain-containing adaptor inducing IFN-β (TRIF), caspase-1 activation, potassium efflux and mitochondria-derived reactive oxygen species (ROS) and DNA (38–41). Moreover, this effect appears to be largely dependent on the NLRP3 inflammasome (38, 40).

**Figure 2:**
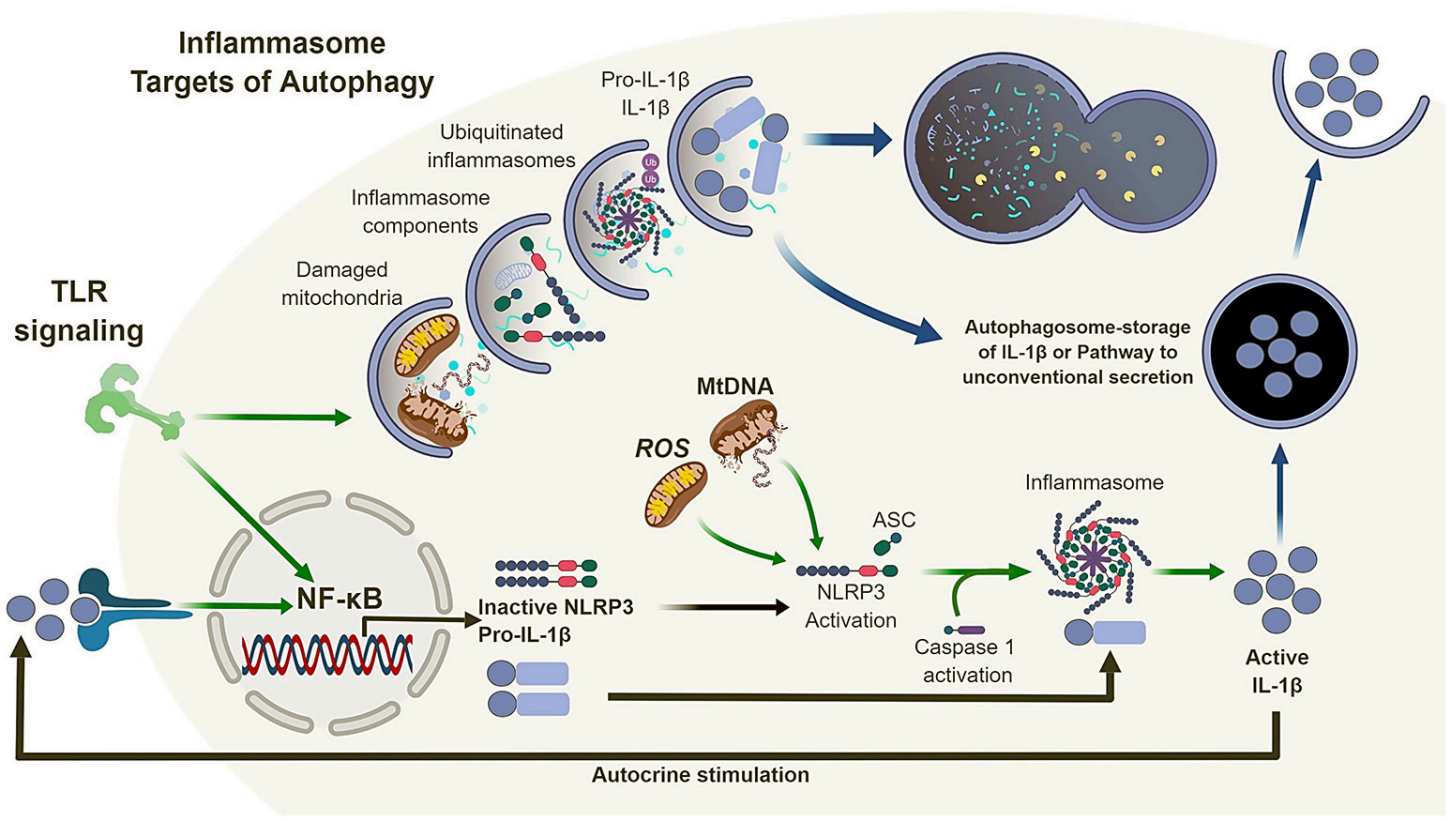
Autophagy and the inflammasome. TLR signaling results in the activation of NF-κB which in turn regulates the expression of NLRP3 and pro-IL-1β. Mitochondrial reactive oxygen species (ROS) and DNA can lead to the activation and oligomerization of NLRP3 and recruitment of the adaptor protein ASC. This complex recruit and activates caspase 1 subsequently, recruiting, and activating IL-1β. Autophagy is also induced by TLR signaling which in turn can regulate the inflammasome pathway via direct clearance of activators (mitochondria), inflammasomes and subunits (NLRP3, ASC) as well as its substrates (pro-IL-1β). In the absence of autophagy, damaged mitochondria can accumulate and activate the inflammasome pathway leading to the dysregulation of IL-1β production and secretion.

Inflammasomes are multi-protein complexes which activate caspase-1 (and in certain cases caspase-4/5) in response to pathogen-associated molecular patterns (PAMPs) and damage-associated molecular patterns (DAMPs) (42–45). The secretion of IL-1β and IL-18 is typically a two-stage process. First, transcription and translation of inactive pro-forms of the cytokines are induced following ligation of pattern recognition receptors, such as TLRs. Second, inflammasome activation occurs in response to ligation or activation of a NOD-like receptor (NLR), such as NLRP1, NLRP3, or NLRC4, or an AIM2-like receptor (ALR) (46). In most cases, the NLR or ALR forms a complex with apoptosis-associated speck-like protein containing a caspase activation and recruitment domain (ASC) to engage and activate caspase-1, which in turn leads to the cleavage and release of pro-IL-1β or pro-IL-18 into mature, bioactive cytokines (Figure 2). The NLRP3 inflammasomes is activated in response to multiple stimuli, including particulates, such as uric acid crystals, vaccine adjuvants and silica (47–49), as well as reactive oxygen species (ROS) and mitochondrial DNA (39, 41).

Thus, loss of autophagy, and more specifically mitophagy (a form of autophagy that sequesters and degrades mitochondria) (50), in macrophages and DC, combined with inflammatory stimulation, can lead to the accumulation of damaged/dysfunctional mitochondria. This chain of events leads to the release of mitochondrial ROS and DNA into the cytosol, which in turns activates the NLRP3 inflammasome, leading to increased caspase-1-dependent processing and release of IL-1β and IL-18. However, the link between autophagy and inflammasome regulation does not end there (Figure 2).

Many studies suggest direct links between autophagy, inflammasomes, and inflammatory signaling pathways, although the complexity of this subject is considerable, due to the numerous means autophagy can regulate the same pathways. For example, regulation of inflammasome activation and release of IL-1 family members by autophagy has an indirect effect on nuclear factor kappa-light-chain-enhancer of activated B cells (NF-κB) pathway, as these cytokines (IL-1, IL-18) all signal through NF-κB- dependent pathways. NF-κB is an evolutionary conserved transcription factor that regulates an ever-expanding network of genes with a wide range of functions in cell survival, differentiation, proliferation, and immunity, acting downstream TLRs and cytokine receptors. Studies have demonstrated that, following activation of NF-κB, autophagy plays a role in the degradation of the NF-κB inhibitor family IκB, leading to persistent activation and inflammation (51). Additionally, autophagy inhibits inflammasome activation and IL-1β release by stimulated macrophages through elimination of mitochondrial products, pro-IL-1β and inflammasome components, which in turns leads to reduced NF-κB activation by IL-1β (38, 52, 53). Conversely, loss of autophagy in macrophages and DC leads to increased IL-1β release which activates NF-κB in an autocrine manner (54). Accordingly, autophagy and NF-κB-dependent inflammation and cytokine release converge on multiple levels in APC, with significant impact on cell function and subsequent modulation of immune responses.

Another important function of autophagy is to clear protein complexes that are too large for proteasomal degradation. One study suggests that active inflammasomes may be targeted for lysosomal degradation by autophagosomes (53). These data would suggest that autophagy might represent an important checkpoint for controlling excessive inflammasome activation, which can lead to inflammatory pathologies. Autophagosomes have also been shown to specifically target IL-1β following activation with TLR agonists (38). However, the fate of this autophagosomal IL-1β is potentially more complex than that suggested for inflammasomes. Induction of autophagy during or after priming of cells with a TLR agonist results in a reduction of intracellular pro-IL-1β (38), but it is unclear whether it is degraded in the autophagosomes, or whether it is transported elsewhere for proteasomal degradation, which appears to be the more common route of degradation for this cytokine (55). However, autophagy may also have a role to play in the secretion of IL-1β. While most secreted proteins have an N-terminal signal peptide that allows them to traffic through the classical secretion pathway, a number of secreted proteins, including IL-1β and IL-1α, lack such a signal peptide and are released by largely undefined mechanisms, termed unconventional secretion. Two studies have demonstrated that autophagy may play a role in the unconventional secretion of IL-1β (56, 57). Together, these studies may indicate that autophagosomes act as temporary repository for IL-1β, which can either be directed to a degradative or secretory pathway, depending on the context and specific stimuli. It is not clear whether IL-18 is also targeted to autophagosomes. While the biological effects of IL-1α are similar to those of IL-1β, its release is independent of inflammasome activation *per se*. IL-1α's biological activity does not require proteolytic cleavage, although it can be cleaved by both the calcium-dependent cysteine protease calpain and by the cytotoxic lymphocyte-derived protease granzyme B (58, 59). Nevertheless, inflammasome activation does enhance IL-1α release, possibly due to increased cell death. As with IL-1β and IL-18, autophagic defects in APC increases the release of IL-1α in response to inflammatory stimuli, including TLR agonists and allergens (38, 60, 61). However, unlike IL-1β, this release is independent of NLRP3, caspase-1 and TRIF (38, 60), but dependent on ROS and calpain (60). It is not yet clear whether autophagy is involved in the unconventional secretion of IL-1α, or whether IL-1α associates with autophagosomes.

### APC Autophagy and the TH17 Connection

A large and ever-growing body of evidence has established multiple roles for autophagy in the regulation of proinflammatory cytokines (IL-1, IL-18) secreted by APC. A disruption in the autophagy pathway has also been shown to impact the secretion of other proinflammatory cytokines (IL-17, IL-23) and chemokines (CXCL1) (54, 60). For example, IL-1β released from macrophages can stimulate IL-23 secretion in an autocrine manner (62) and this occurs in LPS-stimulated macrophages and DC in which autophagy is inhibited (54). Secretion of IL-23 under these circumstances can be inhibited with an IL-1 receptor antagonist or neutralizing antibody against IL-1β. Similarly, autophagy-deficient macrophages that secrete excess IL-1α have increased CXCL1 output that can be curbed by an IL-1 receptor antagonist (60). Conversely, inducing autophagy after *in vitro* APC stimulation reduces IL-1α, IL-1β, IL-23, and CXCL1 secretion (54, 60). Additionally, treating mice with the autophagy inducer rapamycin inhibits both IL-1β and IL-23 in response to intraperitoneal injection of LPS (38, 54).

Whether directly or indirectly, autophagy appears to regulate cytokines and chemokines that promote IL-17-mediated immune responses. The combination of IL-23 with IL-1α/IL-1β, promotes TH17 cellular differentiation and stimulates the secretion of IL-17 from innate-like γδ T cells (63–65). IL-1, IL-17, and CXCL1 can cause neutrophilic tissue infiltration (66–68). Furthermore, *in vitro* treatment of naïve murine CD3^+^ T cells with supernatants from LPS and 3-MA-treated dendritic cells (high in IL-1α, IL-1β, and IL-23), enhances secretion of IL-17A, IL-17F, IFN-γ, and IL-22 (54).

**Figure 3:**
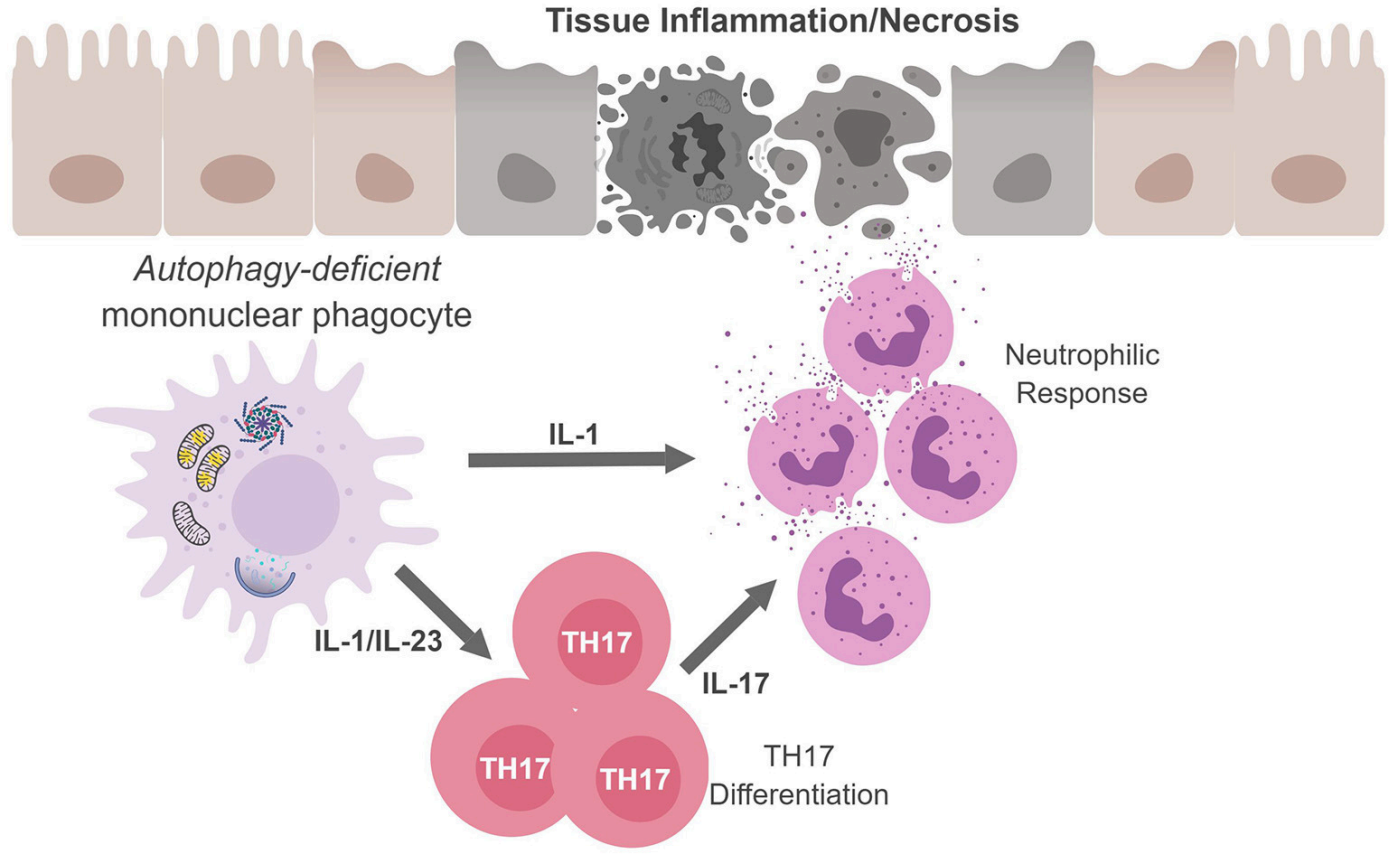
Mononuclear phagocytes cell-autonomous autophagy curbs TH17 responses. Autophagic defects in mononuclear phagocytes results in the accumulation of damaged/dysfunctional mitochondria. Inflammatory activation of mononuclear phagocytes can increase mitochondrial dysfunction and the release of mitochondrial ROS and DNA that activates the inflammasome. Inflammasome activation results in excess IL-1β production and secretion. IL-1β can have (i) autocrine effects and induce IL-23, (ii) IL-1β and IL-23 can drive TH17 differentiation; and (iii) along with IL-17, IL-1β can recruit neutrophils. The inability to curb inflammasome activation due to an autophagic defect can result in continuous inflammation and tissue damage.

Regarding TH17 cells, these CD4^+^ T cells play an important role in controlling extracellular bacteria and fungi by producing the cytokines IL-17A and IL-17F that act on epithelial cells to recruit neutrophils. TH17 cells and cytokines associated with TH17 responses have also been implicated in numerous autoimmune and infectious diseases (69, 70). In fact, several studies confirm that autophagy disruption in APC during infection leads to excessive inflammation which is associated with upregulated levels of IL-1 and IL-17 (Figure 3) (60, 61, 71, 72). Given the loss of autophagy in APC leads to increased secretion of IL-1 family cytokines and IL-23 upon inflammatory stimulation, it is perhaps not surprising that mice with autophagy-deficient myeloid cells (*Atg5*^fl/fl^-LysM Cre mice) show elevated serum IL-17 in response to infection with *Mycobacterium tuberculosis* (60). Similarly, mice deficient in the autophagy protein LC3B (MAP1-LC3B) exhibit increased IL-17-induced lung pathology upon infection with respiratory syncytial virus (RSV) and *Map1lc3b*^−/−^ CD11b^+^ DC infected with RSV induce IL-17 secretion from CD4^+^ T cells in an IL-1-dependent manner (72). Moreover, *Atg5*^fl/fl^-CD11c Cre mice develop spontaneous airway hyperreactivity and severe neutrophilic lung inflammation, with elevated IL-1 and IL-17 levels in the lungs (61). Together, this points to autophagy as a key regulator of TH17 immune responses.

### Autophagy and APC-Derived MIF

Similar to IL-1 family cytokines, loss of autophagy in human and mouse macrophages leads to increased secretion of macrophage migration inhibitory factor (MIF) in response to LPS (73). MIF is expressed in multiple immune cell types, including macrophages, DC, T, and B cells, neutrophils, eosinophils, and mast cells. MIF is critical in the innate immune response to different bacteria, including *Salmonella* and *Mycobacterium* species. MIF can also upregulate TLR4 and increase the release of other pro-inflammatory cytokines, including TNF-α, IL-1, and IFN-γ (74). Interestingly, MIF also directly regulates NLRP3 inflammasome activation, facilitating the processing and release of IL-1β and IL-18 (75). The regulation of MIF by autophagy is dependent on mitochondrial ROS, which accumulates in the cytosol of autophagy-deficient cells. Amino acid starvation, an inducer of autophagy, also results in increased MIF secretion (73, 76). Given that amino starvation is a strong inducer of autophagy, these results may seem contradictory. However, MIF release is unaffected by other autophagy inducers (mammalian target of rapamycin; mTOR inhibitors) (73), suggesting that autophagy induction through mTOR inhibition is not responsible for MIF release. Concerning MIF and T cell activation, MIF has also been shown to induce IL-17 expression and secretion in mouse lymph nodes (77). Moreover, in a mouse model of gout, MIF levels are raised, and MIF deficiency or blockade lowers levels of IL-1β and reduces neutrophil infiltration and pathology (78). Thus, MIF might represent another connection between autophagy, inflammasome activation, and TH17 responses.

### Autophagy and a Possible Role in Controlling TH1 Responses

The above observations suggest functional autophagy may curb detrimental IL-17-mediated inflammatory disorders. Nonetheless, it needs to be pointed out that the exacerbated IL-17 response observed in autophagy-deficient APC animal models could be due to compartmentalization of the infection to the lung. In the intestine, autophagy, specifically in epithelial cells, has proven to be important to maintain intestinal homeostasis (79–83). Additionally, an underappreciated role for autophagy in intestinal mononuclear phagocytes has been established. Saitoh et al. first showed hematopoietic cells lacking *Atg16l1* (through the generation of bone marrow chimeras) succumbed to colitis induction demonstrating autophagy in hematopoietic cells controls intestinal inflammation (40). More recently, mice with autophagy-deficient myeloid cells (*Atg7*^fl/fl^-LysM Cre and *Atg16l1*^fl/fl^ -LysM Cre mice) but not CD11c-expressing innate cells (*Atg16l1f* ^fl/fl^-CD11c Cre mice) displayed enhanced inflammation of the colon after colitis induction with heightened IL-1β levels found in the serum and being produced by macrophages (84–86). Interestingly, Lee et al. found colonic lamina propria T cells from *Atg7*^fl/fl^-LysM Cre mice displayed robust TH1 skewing (IFN-γ production) with no difference in TH17 cells (IL-17 production) after colitis induction (84). IL-12p70, a potent inducer of IFN-γ, was not assessed by either group; however, IL-1β can synergize with IL-12p70 to induce IFN-γ (87–90). Interestingly, enhanced IL-12p70 secretion was reported in macrophages deficient in *Atg5* as was serum IL-12p70 during TB infection in *Atg5*^fl/fl^-LysM Cre mice (60). But it remains unclear if autophagy directly regulates IL-12p70 production or if this an IL-1 autocrine effect as seen with IL-23 (54). And while cytokines involved in TH1 responses (IFN-γ and TNF-α) can affect the autophagy pathway, the intersection of autophagy and APC-derived cytokines driving TH1 (IL-12p70) responses need to be investigated further (91–96).

Past studies have revealed the importance of IFN-γ responses in enteric infections. Specifically, IFN-γ-deficient mice were shown to be susceptible to *Citrobacter rodentium* infection as were IL-12p40-deficient mice both demonstrating IFN-γ responses are critical for *C. rodentium* infections (97, 98). IL-12p40 is a protein subunit that dimerizes with IL-12p35 to form the heterodimeric cytokine, IL-12p70 or IL-23p19 to form IL-23 (90). Regarding enteric infections and autophagy, the Cadwell lab revealed ATG16L1 hypomorph mice were resistant to *C. rodentium* infection albeit in the absence of CD4^+^ T cells (99). It is unclear if this resistance is due to an exacerbated IFN-γ response by innate lymphocytes. Several studies have shown the influence of lamina propria IFN-γ-producing innate lymphocytes in intestinal infections and IBD (100–102). If IL-12p70 production is dysregulated in autophagy-deficient APC this could lead to enhanced IFN-γ response by T cells and innate lymphoid cells and resistance to *C. rodentium* infection. It could also explain human diseases associated with autophagy defects and strong TH1 and TH17 responses such as IBD, MS, and RA (103–105). Collectively, these studies highlight the impact autophagy may have on skewing immune responses and suggest the microenvironment could ultimately determine how autophagy controls TH cell polarization.

## Cell-Autonomous Autophagy Governs T Cell Biology

**Figure 4:**
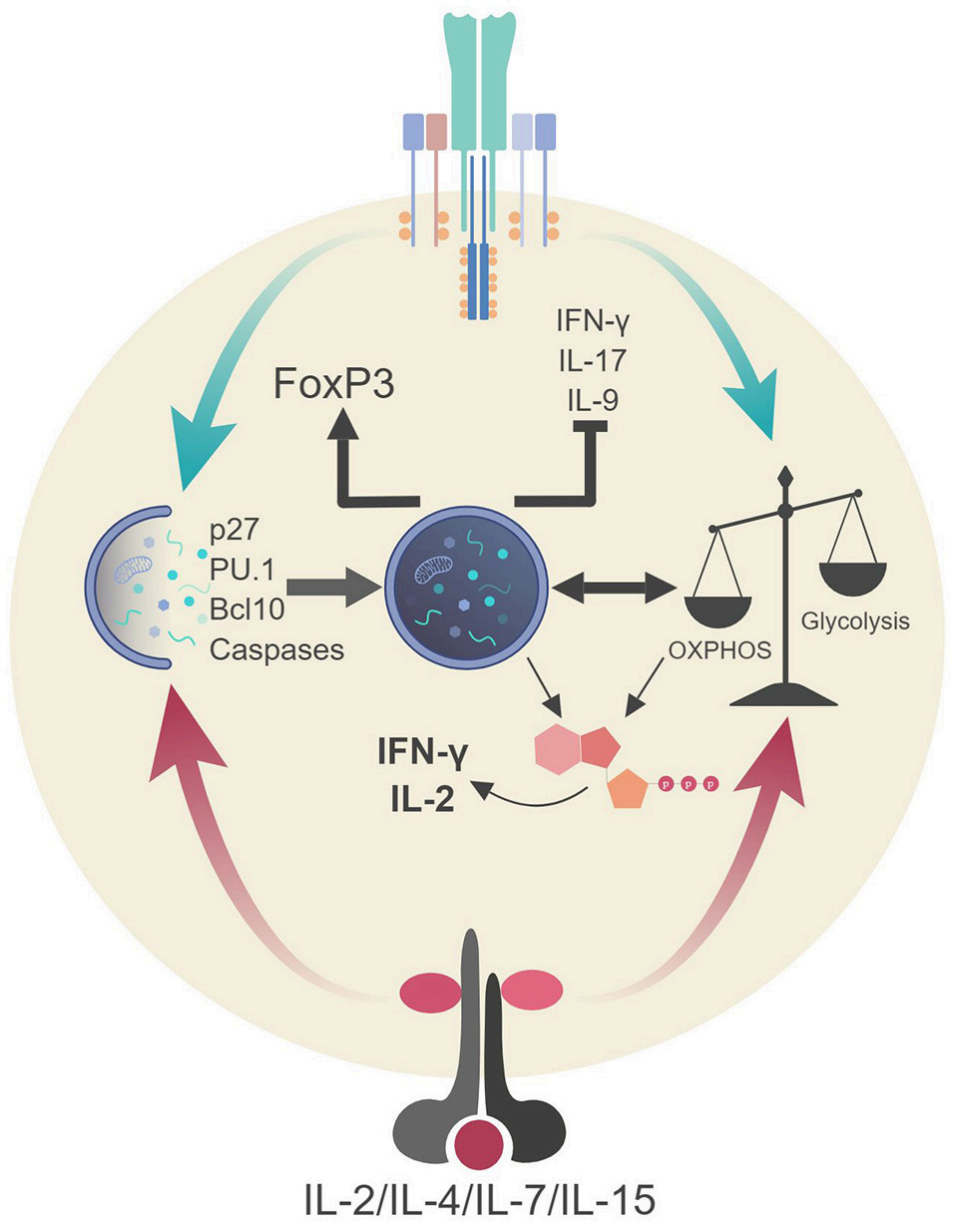
Autophagic regulation of T cell function and metabolism. Autophagy activation via TCR stimulation (and possibly IL-2 production) assists in the proliferation and survival of T cells through direct targeting of p27 (cell cycle inhibitor) and pro-apoptotic factors (i.e., caspases, Bim). Autophagy also targets Bcl10 for degradation to limit NF-κB activation. Additionally, autophagy and metabolic pathways are activated via TCR stimulation and γc-cytokine signaling. These pathways can communicate to generate ATP in TH1 cells which assists in the production of IL-2 and IFN-γ. Autophagy in Tregs impedes glycolysis which stabilizes the regulatory phenotype by maintaining FoxP3 expression and inhibiting the transcriptional network of effector T cells. Lastly, autophagy inhibits TH9 differentiation and subsequent effector function (IL-9 production) via selective targeting of the TH9 lineage specific transcription factor, PU.1.

As discussed above, autophagy affects APC function in a way that can modulate T cell responses. Similarly, autophagy is quite functional in both CD8^+^ and CD4^+^ T cells from naïve to memory cellular states. Mature naïve T cells display a very small, but detectable, level of basal autophagy but are also capable of up-regulating autophagy in response to multiple stimuli. This autophagic program appears to regulate survival and proliferation as well as organelle quality control. Effector and memory T cells both utilize an autophagic program that interacts with intracellular metabolic pathways. This intersection of autophagy and metabolic pathways assists in T cell differentiation and functional capabilities. Through extensive work in genetics, various groups have shown autophagy is an active pathway involved in numerous aspects in T cell biology (Figure 4) as discussed below.

### Autophagy Regulates T Cell Survival and Proliferation

Over the last decade, several groups have confirmed autophagy as an active cellular process in T cells (106–112). Initially, the confirmation of this pathway, detection of autophagosomes and up-regulation of autophagy proteins, was only observable in T cells after T cell receptor (TCR) stimulation. It was further substantiated to be an active process in naïve T cells due to the expression of numerous autophagy genes (*Atg5, Atg7, Atg8*, and *Beclin-1*) found prior to stimulation. To further understand the role of autophagy in T cells, several groups generated numerous T cell-specific conditional knockout mice to delete various autophagy genes (i.e. *Atg3, Atg5, Atg7, Atg16L1, Beclin-1, Vps34*) involved at difference steps of the autophagy pathway (Figure 1) (106–113). Nevertheless, no matter the autophagy gene deleted, the results were similar between the conditional knockout mice: a reduction in the frequencies of thymocytes as well as peripheral CD8^+^ and CD4^+^ T cells. Also, the T cells present in the periphery of these mice displayed a memory-like phenotype likely due to lymphopenia-induced proliferation (114–117). Additionally, autophagy-deficient T cells showed enhanced apoptosis which was linked to enhanced expression of pro-apoptotic molecules pro-caspase-3, caspase-8, and -9 as well as Bim (106, 113). These early studies pointed to autophagy as a key regulator of T cell homeostasis.

The survival of peripheral T cells is an active and involved process for both naïve and memory T cells. Naïve T cells require continuous MHC-TCR interaction as well as cytokine signals from the tissue microenvironment whereas memory T cells require only the latter (118). IL-7 and IL-15 are two common γ-chain (γc) receptor cytokines important for both naïve and memory T cell homeostasis (119). Interestingly, both IL-7 and IL-15 activate autophagy in T cells along with γc cytokines, IL-2, and IL-4, highlighting an emerging role for the JAK-STAT pathway in regulating autophagy (120). Nevertheless, autophagy appears to be important in long-term T cell homeostasis as IL-7 can maintain autophagy-deficient naive T cells in short-term cultures. In long-term cultures (over 24 days), *Atg3*^−/−^ CD4^+^ and CD8^+^ T cells exhibited a higher rate of apoptosis compared to autophagy-proficient T cells (110). Supporting these findings, another study revealed mature T cells lacking *Atg7* quickly undergo apoptosis despite being in conditions with high levels of pro-survival homeostatic cytokines (121). These *Atg7*^−/−^ T cells also failed to efficiently proliferate in response to TCR stimulation (121). T cells lacking *Atg3, Atg5*, or *Vps34* also exhibit proliferation defects (107, 112, 122) which appear to be dependent on the accumulation of Cyclin-dependent kinase inhibitor 1B (CDKN1B/p27/Kip1) which controls the cell cycle progression at G1 by inhibiting the activation of cyclin E-CDK2 and cyclin D-CDK4 complexes. Consequently, demonstrating autophagy provides numerous non-redundant mechanisms to ensure the survival and proliferation of T cells beyond homeostatic cytokine signaling and constant TCR-to-MHC contact that is required for both naive and memory T cell homeostasis.

### Autophagy Is Essential for CD4^+^ T Cell Differentiation

Naïve CD4^+^ T cell differentiation is an intricate process involving interaction with professional APC. T cell activation and differentiation is driven by two simultaneous events—signal (1) MHC II-Ag/TCR interaction and signal (2) co-stimulation (e.g., CD80/CD86–CD28). In addition, APC-derived cytokine signaling assists in lineage commitment. These events trigger a signaling cascade that induces proliferation as well as differentiation that is mediated by the expression of lineage-specific transcription factors (123). This differentiation process grants CD4^+^ TH cell subsets with specific effector functions. In fact, the differentiation of each TH subset is driven by a specific cytokine like IL-12 for TH1 cells, IL-4 for TH2 cells and TGFβ for regulatory CD4^+^ T cells (Treg) (123). Other TH subsets require a combination of cytokines as is the case for TH17 cells which require TGF-β, IL-1, and IL-6 whereas TGF-β and IL-4 drives TH9 differentiation (123). The outcome allows each CD4^+^ T cell effector subset (TH1, TH2, TH9, TH17) to play an important role in activating both innate and adaptive arms of the immune system to specific pathogens. In addition, differentiation can lead to immunosuppressive functions as is the case for Tregs. Both effector and regulatory CD4^+^ T cells play an important role in host immunity, and a defect in these pathways are linked to numerous inflammatory and autoimmune diseases (123).

Given that TCR and cytokine stimulation can activate autophagy it is easy to speculate autophagy has a hand in CD4^+^ T cell differentiation and function. However, autophagy's influence on T cell differentiation and function has remained unclear for many years due to autophagy's critical role in T cell survival (107, 112, 124, 125). Moreover, it appears there is a varying degree to which each subset of CD4^+^ T cells utilize autophagy for differentiation and function. For example, autophagy appears to have an inhibitory effect in the expansion and differentiation of both TH2 and TH9 cells (126–128). Whereas, TH1 and Treg cells appear to rely heavily on autophagy for differentiation and function (121, 126). This discrepancy could be due to cytokine signaling pathways utilized by each CD4^+^ T cell subset as well as their respective metabolic phenotype (discussed in the next section).

In CD4^+^ T cells, cytokine signaling plays a major role in regulating both T cell differentiation and function. For instance, IL-2 is secreted immediately after TCR engagement and acts in autocrine fashion. This is crucial for sustained proliferation, survival and effector differentiation. As mentioned above, IL-2 can induce autophagy, thus, there is a high probability CD4^+^ T cell differentiation and function may stem from cytokine-induced autophagy activation (120, 129). Interestingly, TH1 differentiated T cells lacking autophagy display defects in IL-2 and IFN-γ production (121). Moreover, there were no reported defects in TCR signaling suggesting autophagy may be required for T cell function (i.e., cytokine production). It is unclear if this functional defect is due to inaccessibility of the cytokine loci(s) for optimal transcription following activation (130) or the inability to generate ATP in autophagy-deficient cells which is required for optimal cytokine production (121). *Il2*^−/−^ naïve T cells also produce low levels IFN-γ upon stimulation in TH1 differentiating conditions demonstrating IFN-γ production is partially dependent on both IL-2 and IL-12 signals (131, 132). Therefore, this phenotype in autophagy-deficient TH1 cells regarding IFN-γ production may be like the one observed in autophagy-deficient APC regarding the overexpression of IL-1 and its influence on IL-23 production (54, 60). It would be interesting to determine the level and function of autophagy and ATP generation occurring in TCR-stimulated *Il2*^−/−^ T cells, specifically, whether IL-2 is required for ATP generation through mitophagy.

Harris (91) reviewed evidence suggesting that pro-inflammatory cytokines including IFN-γ, TNF-α, IL-1, IL-2, and IL-6 induce autophagy, while anti-inflammatory cytokines IL-4, IL-10, and IL-13 inhibit autophagy (5, 36, 133, 134). Recent reports, however, suggest that autophagy may be induced by various components of antagonistic immune signaling pathways. For example, IL-10 has been shown to promote mitophagy in macrophages (135) and IL-4 promotes autophagy during DC differentiation (136). Cytokine-induced autophagy is therefore likely to be cell- and context-dependent. IL-4, a potent inducer of TH2 differentiation, induces autophagy in DC; however, autophagy appears to hamper TH2 differentiation. Another example is with TH9 cells, whereby autophagy activation is not required for differentiation and in fact inhibits lineage commitment (128). TH9 cells are a subset of CD4^+^ T cells that secrete the proinflammatory cytokine IL-9 and can contribute to antitumor immunity. Additionally, TH9 cells (IL-9 expression) increase during chronic inflammatory diseases like CD (123). TH9 lineage commitment and IL-9 expression are controlled by the transcription factor and master regulator of TH9 cell differentiation, PU.1. Interestingly, PU.1 is selectively targeted for autophagic degradation upon TH9 differentiation (128). A combination of TGF-β and IL-6 stimulation is required for TH9 differentiation and both cytokines can induce autophagy (91, 133). It is still unclear how PU.1 expression/TH9 cells are maintained but autophagy clearly impacts the stability of this subset of CD4^+^ T cells.

Recently, new targeted deletion of autophagy genes in T cell subsets (i.e., CD4-Cre, FoxP3-Cre), has provided clues into autophagy role in the stability of other TH lineages (126, 137, 138). Genetic deletion of *Atg5, Atg7, or Atg16l1* in Foxp3-expressing T cells results in multi-organ inflammation in mice. There was still a dramatic decrease in Treg cells due to enhanced apoptosis. However, one study found a defective autophagic pathway in Tregs leads to destabilization of the Treg phenotype and loss of suppressive function (137). It was suggested the Treg phenotype was in peril due to a decrease in expression of the lineage-specific transcription factor Foxp3 and enhanced TH1/TH17 effector function (IFN-γ/IL-17 production). It is unclear if this is a direct effect of autophagic function or systemic inflammation as inflammatory stimuli destabilizes FoxP3 expression in Tregs (139). Moreover, whether FoxP3 is a target for autophagic degradation like PU.1 is unclear but these studies suggest autophagy may play a role in lineage stabilization. Nevertheless, further investigation is necessary to definitively demonstrate that autophagy is required for Foxp3 stabilization and Treg function independent of autophagy's impact on Treg survival.

To conclude, these studies shed new light on the autophagic pathway in T cell differentiation. But they also emphasize new possibilities with regards to how autophagy is induced especially along with other signaling pathways. For instance, TCR signaling can activate both autophagy and mammalian target of rapamycin (mTOR) complex nutrient sensing pathways (121, 131). So how does a differentiating T cell deal with these opposing pathways on top of cytokine stimulation? Additionally, are specific autophagic functions being induced by various TH-differentiating cytokines, and do these functions differ from the canonical degradative autophagic pathway? Similar to TH9 cells and possibly Treg cells, does autophagy degrade other TH lineage specific transcription factors? To decipher the numerous autophagic functions in T cells, new sophisticated genetic models are required to understand the complexity of TH differentiation. To add further complexity to these questions, we are finding metabolism is influential in T cell function and that autophagy and metabolism are interconnected as we discuss below. A complete understanding of these intricate details could provide clues to the next therapeutic agents to modulate T cell responses via autophagy.

## Convergence of Autophagy and Metabolism to Control Immune Function

Recent evidence has highlighted the importance of intracellular metabolic programming in immune cells (140, 141). This metabolic change between aerobic glycolysis and oxidative phosphorylation (OXPHOS) impacts both the innate and adaptive arms of the immune system, particularly the function and differentiation of cells. These metabolic pathways are triggered by environmental cues such as nutrients, O_2_ levels, and activation state, which are each modulated by numerous stimuli. Moreover, autophagy has been shown to affect these metabolic pathways (142).

A functional autophagic pathway is frequently required for successful metabolic reprogramming during differentiation states of immune cells. For competent immunogenic (or homeostatic) cellular differentiation, both metabolism and autophagy must accurately sense nutrient levels and respond such that nutrients and biomolecules are appropriately utilized, stored, or recycled (5, 7, 143). For instance, induced shifts between different metabolic and autophagic profiles in immune cells are often regulated by shared networks of nutrient-sensing pathways, such as the mammalian target of rapamycin complex 1 (mTORC1) and AMP-activated protein kinase (AMPK) axes (144–146).

Evidence from various immune cells converges around two distinct inducible profiles of metabolic signaling and function: one for an “active-state” and another for the “steady-state” (140, 144–146). This evidence is summarized in Table 1. The kinase mTOR is a potent negative regulator of autophagy, and its associated mTORC1 complex is known to play a role in inducing a metabolic active-state (145–147). AMPK, conversely, triggers ATP generation via fatty acid oxidation (FAO), promoting a metabolic steady-state. Additionally, AMPK indirectly and directly facilitates autophagy by suppressing mTORC1 activity and activating autophagy-initiating kinase ULK1 (148).

**Table 1:** Active and steady state metabolic profiles of immune cells.

	**Active state**	**Steady state**
Metabolic polarization axis	M1 macrophages	M2 or quiescent macrophages
	Activated DC	Quiescent or Naïve DC
	TH1	Naïve mature CD4^+^ T-cells, CD4^+^ Memory T cells
	TH2	TH2, Naïve mature CD4^+^ T cells, CD4^+^ memory T cells
	TH17	CD4^+^ Treg, CD4^+^ memory T cells
	Activated CD8^+^ T cells	Naïve mature CD8^+^ T cells, CD8^+^ memory T cells
Bias of metabolic profile	Anabolism: aerobic glycolysis, glutaminolysis, fatty acid synthesis, pentose phosphate pathway	Catabolism: oxidative phosphorylation, fatty acid oxidation
Upstream activated nutrient sensing pathway	mTOR	AMPK
Nutrient sensing pathway's effect on autophagic profile	mTOR: inhibits autophagy via inhibiting ULK1 activity	AMPK: stimulates autophagy via activating ULK1 and inhibiting mTOR

*This table provides a general overview of immunometabolism in various immune subsets including APC and CD4^+^ and CD8^+^ T cells*.

An active-state metabolic profil*e* is generally anabolic, relying on aerobic glycolysis, glutaminolysis, fatty acid synthesis (FAS), and/or the Pentose-5 Phosphate Pathway (PPP) to generate amino acids, nucleotide precursors, redox co-enzymes, and membrane lipids to facilitate immunogenic functions (149–151). The steady-state metabolic profile, on the other hand, relies on the more sustainable and efficient ATP-generation capacity of catabolic pathways such as OXPHOS and FAO (140, 148, 149, 151). A growing literature suggests many hallmarks for active- versus steady-states are conserved across hematopoietic and non-hematopoietic cell-types (7, 140, 146, 149).

Autophagy plays numerous roles in basic cellular function during both “active” and “steady” metabolic states across immune cell-types, including for immunogenic function during antigen presentation, cytokine secretion, and regulation of inflammation (152–155). Surprisingly, autophagy is induced by various components of antagonistic immune and metabolic signaling pathways. For example, anti-inflammatory cytokines such as IL-10 and IL-4 appear to promote both steady-state metabolism and autophagy (135, 156–158), while IL-2, TGF-β, and IFN-γ promote the metabolic active-state as well as induce autophagy (133, 134). Nevertheless, the influence autophagy and these metabolic pathways have on immune cell function highlights potential therapeutic targets to modulate T cell responses. Below we discuss the interconnection between autophagy and metabolism in both APC and T cells.

### Cross-Talk Between Autophagic and Metabolic Pathways in Antigen-Presenting Cells

Studies have shown that macrophages and DC undergo profound metabolic changes in response to activation. For instance, stimulation of macrophages with lipopolysaccharide (LPS) leads to a shift in metabolism toward increased glycolysis, while IL-4 promotes OXPHOS (156–158), suggesting that metabolic profiles may influence, or are influenced by, macrophage polarization. More recently, Ip et al.^.^reported the loss of IL-10-responsiveness in macrophages increased glycolysis and lowered OXPHOS in response to LPS treatment which promoted a proinflammatory phenotype (135). They went on to show that IL-10 inhibits glycolysis by reducing LPS-induced glucose uptake and downregulating glycolytic gene expression. Moreover, the reduced OXPHOS in LPS-treated *Il10*^−/−^ macrophages was accompanied by an increase in dysfunctional mitochondria, suggesting that IL-10 is required for mitophagy during activation to limit proinflammatory responses in macrophages (135).

The mechanism by which IL-10 induces autophagy is not fully understood, but IL-10 was shown to regulate mTOR through STAT3-dependent upregulation of the mTOR inhibitor DDIT (135). IL-10 may simultaneously regulate autophagy/ mitophagy through activation of AMPK (135, 159). Another study has shown that LPS induces the expression of interferon regulatory factor-1 (IRF-1), which increases mitochondrial damage (160) and inhibits macrophage mitophagy (161, 162). It is not fully clear whether IRF-1 directly regulates IL-10, or vice versa, but one study has demonstrated that *Irf1*^−/−^ DC express higher levels of IL-10 (163), suggesting a possible connection. Other studies have suggested that IL-10 inhibits autophagy in response to starvation, rapamycin and IL-17 via both STAT3 and Akt signaling pathways (164–166). Whether IL-10 can exert different effects on autophagy depending of stimulus or context, or whether it specifically regulates mitophagy, rather than other forms of autophagy, remains to be elucidated.

Interestingly, *Il10* polymorphisms confer increased risk for CD as well as autoimmune diseases SLE, RA and MS (167–170). Additionally, defects in IL-10 signaling specifically in intestinal macrophages disrupt an educational process that differentiate macrophages to become anti-inflammatory and tolerant (i.e., limiting proinflammatory cytokine secretion) to microbial stimulation; and a loss of this educational process leads to colitis in mice (171–173). Moreover, studies have indicated that autophagy may influence the polarization of other tissue macrophages. Notably, autophagy promotes cell survival during monocyte-macrophage differentiation (174, 175) and loss of autophagy appears to promote differentiation of M1 macrophages and decreases their potential to differentiate into M2 macrophages (176–178). This, in turn, will greatly affect the response of those macrophages to stimuli, particularly their cytokine profile, which has the potential to change the resulting T cell response. In the most simplistic terms, M1 polarization of macrophages will favor TH1 polarization of T cells, while M2 macrophages (particularly M2a and M2b) promote TH2 responses (179). Nevertheless, as discussed above, defects in the autophagy pathway in macrophages or DC tend to drive a hyperresponsive IL-17/TH17 response (Figure 3).

### Autophagy and T Cell Metabolism

Initial stimulation of TCR leads to the activation of both mTOR and mTOR-independent autophagy nutrient sensing pathways. Autophagy is possibly induced by a JAK/STAT pathway downstream of IL-2 and IL-4 signaling (120). Induction of autophagy in CD4^+^ T cells by other common γc cytokines such as IL-7 and IL-15 may act through the same pathway. These signals initiate complete metabolic reprogramming in activated T cells. This reprogramming is also associated with a global change in the metabolic transcriptome, with induction of endogenous myelocytomatosis oncogene (c-Myc) and hypoxia inducible factor 1, alpha subunit (HIF1α). The mTORC1-c-Myc pathway has been demonstrated to directly regulate T cell proliferation through transcriptional control of cell cycle regulators (180, 181), and has been implicated as an essential coordinator of T cell activation-induced cell growth and proliferation (182). HIF1α and c-Myc are both critical for the upregulation of glucose transporters and glycolytic enzymes, and at least some of mTOR's pro-glycolytic actions are mediated by its upregulation of HIF1α (183, 184).

Interestingly, autophagy-deficient CD4^+^ T cells show increased expression of c-Myc and a glycolytic phenotype suggesting autophagy inhibits glycolysis. In Tregs, this loss of autophagy and subsequent enhanced glycolytic metabolism results in lineage destabilization and loss of effector function (126, 137). Although this an extreme scenario, these studies demonstrate (i) metabolism dictates effector function in T cells and (ii) autophagy is a major regulator of metabolic profiles. T cells generally conform to the “active” and “steady-state” metabolic profile dichotomy, though the unique demands of T cell immunogenic functions give rise to distinct metabolic trajectories between subsets (7, 149, 185). For example, Lunt and Vander Heiden (149) report that the shift toward glycolytic anabolism during the active state supports the needs of proliferation across cell types by generating the materials required to produce new daughter cells (149). Activated T cells have been shown to rely on OXPHOS to support early proliferation and cytokine production. Glycolytic-incompetent T cells can proliferate upon activation whereas OXPHOS-incompetent T cells fail to do so (144). Consequently, activated T cells appear to maintain mitochondrial ATP generation via OXPHOS for between 1 and 2 days after activation in part to support the metabolic demands of proliferation (144, 185). ATP generation via OXPHOS is also required for efficient IFN-γ production by TH1 cells. A disruption in autophagy (a catabolic process) diminishes ATP and IFN-γ output from TH1 cells (121). Furthermore, glycolytic-incompetent differentiated CD4^+^ T cells, while able to proliferate, exhibit a diminished effector response, mediated in part by GAPDH's competitive role as a negative regulator of IFN-γ mRNA in the absence of glycolytic metabolism (144).

An overview of active and steady state profiles of T cells are provided in Table 1. Activated and differentiated T cells are highly anabolic, while naïve and memory T cells are overall more catabolic. Evidence also supports further nuance in metabolic phenotypes of other TH subsets. For example, TH17 cells appear to rely more heavily on fatty acid synthesis than do other effector T cell subsets (186), and TH2 cells are more dependent on glycolysis for effector function than are Tregs, TH1, or TH17 cells (126). In fact, autophagy (and its inhibitory role in glycolysis) appear to restrict TH2 expansion. Several of TH2 effector functions are also in part dependent on FAO and OXPHOS (187, 188). This dual nature of TH2 metabolism is represented by its placement in both “active” and “steady” states. Additionally, AMPK has been shown to support CD8^+^ T cell effector function in glucose-starved conditions while FAO is needed for the transition from effector to memory CD8 T cell (145, 148, 151, 185–187, 189).

Discerning the degree to which different autophagic pathways coincide with, negatively regulate, or enhance polarization toward active- or steady-state metabolic pathways remains a considerable challenge. Still, evidence suggests that some major autophagic pathways may more readily co-occur with the catabolic steady-state, an effect in part mediated by AMPK (145, 146, 148). Future therapeutic and research programs will benefit from the consideration of the substantial cross-talk between metabolic and autophagic signaling pathways. Additionally, treatments targeting autophagy should include holistic consideration of the treatment's effect on the metabolic and profile of the target cell-type compared to the known profile for cells with the desired therapeutic effector or suppressor function.

## Therapeutic Targeting of Autophagy to Modulate T Cell Responses

Recently, there has been surge of interest in examining the therapeutic potential of autophagy in various human diseases (190). In fact, numerous clinical trials are on-going with autophagy modulating agents for both neurodegenerative diseases and various cancers (190, 191). This tremendous attention is supported by both animal models and clinical conditions demonstrating alterations in the autophagic pathway is linked to these human pathological conditions. Besides neurological diseases and cancer, autoimmune diseases and metabolic disorders have also been linked to autophagy (192, 193). Additionally, there has been considerable interest in discovering novel agents as well as repurposing FDA-approved pharmaceuticals to modulate autophagy (194–196). Below we discuss potential T cell-linked diseases that may benefit from autophagy modulation.

### Inflammatory Bowel Disease

Inflammatory bowel disease (IBD) is a chronic relapsing inflammatory response of the gastrointestinal tract, and CD and ulcerative colitis (UC) are its major forms (197, 198). There are several underlying causes of IBD including immune dysregulation, intestinal barrier defects and dysbiosis, which all pose a significant barrier for clinicians. Dysregulated T cell responses including TH1 (IFN-γ) and TH17 (IL-17) cells also appear to contribute to IBD pathogenesis (103); however, an elusive goal in the field of IBD is to identify the causative factors initiating and maintaining chronic inflammation. Furthermore, this diversity of physiological defects along with the numerous cell-types (T cells, APC and epithelial cells) and cytokines (IL-1, IL-2, IL-6, IL-12, IL-13, IL-17, IL-23, IFN-γ, and TNF-α) involved in inflammation make treating IBD a daunting task (199, 200).

Currently, there are five main categories of therapeutics utilized to treat IBD patients: aminosalicylates, corticosteroids, immunomodulators (e.g., azathioprine), antibiotics (e.g., ciprofloxacin and metronidazole), and biological therapeutics (201). Biologics currently show the most potential for patients with moderate to severe CD and UC (202, 203). As mentioned above proinflammatory cytokines are highly expressed during IBD and are a current target for biological therapeutics. One biologic, infliximab, an antibody targeting TNF-α, a cytokine highly elevated in IBD patients, has had some clinical success (204, 205). At least 40% of CD patients and 25% of UC patients go into clinical remission when treated with infliximab alone. When used in combination with the immunomodulator drug azathioprine up to 56% of CD patients go into clinical remission (205). Regarding T cells, vedolizumab, a monoclonal antibody against α4β7 integrin which should prevent T cell binding to MAdCAM-1 and entry into the intestinal mucosa has had some clinical success in patients with moderate to severe CD and UC with clinical remission rates up to 39% and 41.8% at week 52, respectively (206, 207). Overall, the best treatment affects a little more than 50% of the subjects, which at best gives patients a 2-3-year period of remission before relapsing and left with no other option but surgery. Therefore, novel therapeutics are urgently needed to offer better and longer-lasting treatment options for IBD patients.

Through the work of many, autophagy appears to be vital for immune regulation, the intestinal barrier and host-bacteria interaction. Not surprisingly, there have been numerous genes identified that are within IBD risk loci that contribute to the autophagic pathway [reviewed in (208)]. The most well-known and studied is the autophagy gene *ATG16L1* which in Crohn's patients is encoded as a missense variant *ATG16L1* T300A (30–33). ATG16L1 functions as a core autophagy factor (Figure 1) and individuals carrying the variant *ATG16L1* T300A display immune dysregulation and intestinal barrier defects (79, 80, 82, 209). Other identified IBD risk loci involved in autophagy include *ULK1* and *MTMR3* or specifically, in xenophagy (a form of autophagy utilized to degrade pathogens) include *IRGM* and *SMURF1* (210–216). Recently, several animal models of colitis that mimic IBD have demonstrated targeting autophagy can prevent intestinal inflammation (217, 218). These autophagy-inducing agents reduced the expression of proinflammatory cytokines and CD4^+^ T cell infiltration into the mucosa *in vivo*. It is likely autophagy induction is also acting on numerous other cells and pathways that contribute to intestinal inflammation. For example, autophagy induction in colonic intestinal epithelial cells enhances barrier function (81, 82, 209, 219). Some of the autophagy modulators utilized in these studies include sirolimus (rapamycin), everolimus (a derivative of sirolimus), and trehalose (a disaccharide). The mode of autophagy induction for sirolimus and everolimus is through mTORC1 inhibition (220). Trehalose induces autophagy through AMPK activation independent of mTORC1 (221). Interestingly, dietary trehalose (commonly used as a food additive) has been shown to exacerbate another intestinal disease caused by the nosocomial bacterium *Clostridioides difficile* by increasing its virulence (222). Nevertheless, these studies provide a proof-of-concept that autophagy can dampen inflammatory responses. Lastly, a recent clinical trial reported targeting the IL-12p70 and IL-23 shared subunit IL-12p40 with the monoclonal antibody ustekinumab induced remission in patients with moderate to severe active Crohn's disease (223). Given the data autophagy may curb both IL-12p70 and IL-23 (via IL-1) production (54, 60), autophagy modulation rather than biological therapy could prove beneficial on many fronts of IBD treatment. This includes dampening inflammation by turning off IL-12p70 and IL-23 that would promote TH1 and TH17 cell differentiation as well as induce xenophagy and enhance the intestinal barrier. Moreover, it would allow the avoidance of issues associated with biologics like immunogenicity and loss of function over time, and the cost associated with maintenance therapy (224).

### Autoimmune and Infectious Diseases

Concerning autoimmune diseases (e.g., RA and MS) and chronic infectious diseases like TB, can autophagy modulation ameliorate disease progression? Targeting autophagy in several animal models of neurodegenerative diseases and TB supports a role for autophagy in ameliorating disease (225). However, these models are assessing autophagy's degradative role in clearing pathogen or disease-causing proteins. Thus, it remains unclear if these therapeutic agents are impacting immune responses, i.e., TH and APC effector and metabolic profiles.

Rheumatoid arthritis (RA) is an autoimmune disease and like IBD is a chronic inflammatory disease except it primary site of inflammation is in the joints (105). IL-17 and TH17 cells have been implicated in early- and disease-onset phase of RA (69, 105) along with T cell plasticity (TH17 to TH1 conversion) and the ability to produce both IFN-γ and IL-17 (226, 227). In addition, unique metabolic profiles of both T cells and macrophages are associated with disease severity reviewed in (228). Furthermore, a variant of the autophagy-related gene *ATG5* has been identified in group of RA patients and protein levels of ATG7 and BECN1 that are involved in autophagy are upregulated in RA patients (34, 229, 230). It is unclear if this variant contributes to the enhancement or inhibition of autophagy as evidence is suggested for both (231, 232) but chloroquine and hydroxychloroquine (inhibitors of autophagy) have shown efficacy in patients with mild to moderate RA (233). Other evidence to support active autophagy in RA pathogenesis comes from the role of autophagy in generating citrullinated peptides which are targeted by autoantibodies and a cause of bone loss (234, 235). Thus, autophagy may act at several levels of disease including the generation and presentation of citrullinated peptides, increased T cell survival, and manipulation of T cell and macrophage metabolism.

MS, a chronic autoimmune disease that attacks the central nervous system is also characterized by potent TH1 and TH17 responses (104). TH17 and IL-17 appear to be involved in both the initiation and maintenance phase of disease for MS (69, 236) and CD4^+^ T cells capable of producing both IFN-γ and IL-17 are present in the disease tissue (104). Similar to RA, autophagy and autophagy genes maybe upregulated in inflammatory cells that contributes to disease pathogenesis. As mentioned above, the autophagy gene *Atg5* was critical in the processing and presentation of self-peptides by dendritic cells to autoreactive T cells suggesting autophagy factors contribute to disease (35, 237). In T cells, enhanced Atg5 levels have been found in patients with MS possibly leading to enhanced survival and an active metabolic state (238, 239). Further evidence for a role of autophagy activation in MS pathogenesis was the identification of a single nucleotide polymorphism in the *CLEC16A* gene (240). CLEC16A regulates MHC II presentation in APC and can regulate autophagy through the modulation of mTOR activity (241, 242). So unlike in IBD (autophagy defects leads to inflammation), hyperactive autophagy appears to contribute to the pathogenesis observed in both RA and MS through T cell survival and metabolism as well as APC antigen presentation.

*M. tuberculosis* infection is the quintessential bacterial model that have been utilized to understand xenophagy (243–247). Additionally, autophagy's role in immune function during TB infection appears to be extremely important for host protection (60, 71). As discussed above, autophagic defects in APC affects cytokine secretion and subsequently promotes an exacerbated TH17-mediated immune response in animal models of TB infection (54, 60, 61, 72). Moreover, polymorphisms in the autophagy-related genes *IRGM, ULK1*, and *P2RX7* are associated with susceptibility to TB infection (243, 248–251). It is unclear if autophagy acts as a double-edge sword controlling TB and the immune response but animal models show the gene *Atg5* in myeloid cells appear to be the most critical for both functions (60, 71, 252).

TH17 cells and TH17-related cytokines are beneficial in the initial phase of TB infections; however, continuous IL-17-mediated inflammation (i.e., neutrophilic infiltration) contributes to TB pathogenesis (70). Thus, curbing TH17-mediated immune responses through autophagy could prove beneficial in TB (60, 70, 252). Furthermore, numerous autophagy inducing agents have been utilized *in vivo* to induce xenophagy to control *M. tuberculosis* infection (225). It could be that the induction of autophagy also limits APC-derived TH17-promoting cytokines (IL-1, IL-23, MIF) and neutrophil recruiting factors (IL-1, CXCL1) as well as influencing TH17 cells toward the catabolic “steady” state. Additionally, whether autophagy has a role in IL-12-TH1/IFN-γ responses remains to be seen but tipping the TH1/TH17/Treg balance could have serious implications in disease progression.

In conclusion, it is clear the complex interconnections between autophagy, immune function and metabolism highlight vital intracellular events that must be coordinated in both T cells and APC to provide protection against pathogens. The last decade has provided novel results demonstrating the importance of autophagy in immune cells with recent research elucidating the interrelationship between autophagy and cellular metabolism. These recent findings highlight how autophagic and metabolic pathways are profoundly intertwined and help determine the balance between health and disease. Autophagy is an attainable therapeutic target; however, numerous details that surround autophagy, metabolism, and immune function as well as the full extent of their crosstalk are still unclear. Therefore, future research must further dissect and understand the many roles autophagy has in immune function and metabolism. Understanding these biological events will have an influential impact on future therapeutic treatments for both autoimmune and infectious diseases.

## Author Contributions

SDM, CJC, XOY, JH, and EFC wrote the manuscript. JH and EFC edited the manuscript.

### Conflict of Interest Statement

The authors declare that the research was conducted in the absence of any commercial or financial relationships that could be construed as a potential conflict of interest.
